# Chemical composition, antioxidant capacity, and thermal behavior of *Saturejahortensis* essential oil

**DOI:** 10.1038/s41598-020-78263-9

**Published:** 2020-12-07

**Authors:** Dorina Rodica Chambre, Cristian Moisa, Andreea Lupitu, Lucian Copolovici, Georgeta Pop, Dana-Maria Copolovici

**Affiliations:** 1 Faculty of Food Engineering, Tourism, and Environmental Protection, Institute for Research, Development, and Innovation in Technical and Natural Sciences, “Aurel Vlaicu” University of Arad, 2 Elena Dragoi St., 310330 Arad, Romania; 2 Faculty of Agriculture, Banat University of Agricultural Sciences and Veterinary Medicine “King Michael 1st of Romania” from Timisoara, 119 Calea Aradului St., 300645 Timisoara, Romania

**Keywords:** Chemical biology, Plant sciences

## Abstract

*Satureja* *hortensis* is one of the representative plants from the *Lamiaceae* family, and its essential oil has been used in various applicative fields, from the food industry to aromatherapy. The changes that occur in heated samples at different temperatures (160, 175, 190 ºC) over different periods (0.5 and 2.5 h) in *Satureja* *hortensis* essential oil composition and chemical properties were evaluated. The results showed that the major chemical composition constituents of the investigated essential oil are *γ*-terpinene + *α*-terpinolene and carvacrol + *p-*cymene and the thermal behavior is dependent on the content. This composition drastically changes through the heating of the samples and causes significant changes in thermal behavior. The present study demonstrated that the concentration of carvacrol in *S.* *hortensis* essential oil is increasing after heating treatment, and the sample heated at 190 ºC for 2.5 h contained more than 91% carvacrol. This simple treatment is a rapid way to obtain carvacrol from the essential oil that has high potential as a natural preservative suitable for the food industry and alternative and complementary medicine.

## Introduction

Medicinal and aromatic plants are important industrial crops (medicinal crops), widely cultivated and distributed in many areas of the world. Usually, they appertain to the *Boraginaceae,* *Gentianaceae,* *Lamiaceae,* *Liliaceae,* *Papaveraceae,* *Rutaceae,* and *Solanaceae* families, and most of them are herbal plants from which important bioactive compounds like essential oils, alkaloids, and polyphenols can be extracted^1^. Essential oils are complex mixtures of natural substances (secondary metabolites) with low molecular weight, secreted by plants to protect them against predators and harsh environmental conditions. They have become an integral part of our everyday life^2,3^.

In the *Lamiaceae* family, volatile oils are outspread, and the use of many species as aromatic herbs and spices has been done since ancient times. Nowadays, apart from the food industry, essential oils have an increasing demand in the production of cosmetics, perfumes, aromatherapy, and holistic medicine^4–6^, being produced worldwide on a large scale (citrus oils, mint, etc.) and on a much smaller scale (agarwood, iris, rose)^2^.

*Satureja* *hortensis* is one of the typical plants of the *Lamiaceae* family, and its essential oil has a wide variation of composition depending on the growing area and climate conditions. As found in literature^5,7–11^ the main components of *S.hortensis* essential oil are carvacrol, *γ*-terpinene, *p*-cymene, thymol, caryophyllene, *⍺*-terpinolene, *β*-pinene, *⍺*-thujene and *⍺*-pinene, which have antibacterial, antiviral, antifungal activities^3,7,8,10,12^. Due to its well-known properties, *S.* *hortensis* essential oil has been imposed in various applicative fields, from the food industry to aromatherapy, which involves several treatments and heating processes that can affect its composition and thermal properties. The data obtained in this paper are meant to complete the previously reported results by other authors related to thermal analysis and kinetics of volatilization for different other essential oils types^13–19^. The importance of the carvacrol, which was determined to be the major constituent of the essential oil of summer savory in the heated samples of essential oil of *S.* *hortensis* (more than 91%), was earlier established and its applications were recently reviewed by Fierascu et al.^20^. Carvacrol is also the major compound of the essential oils obtained from thyme, oregano, pepperwort, wild bergamot. Carvacrol presents antioxidant, antimicrobial, and antiparasitic properties, as well as anti-inflammatory, antinociceptive, hepatoprotective, anticancer, pain management activity^20,21^. Due to these properties, carvacrol is used in the food industry, the beverage industry, and perfumery^20–22^. Mohtashami et al.^23^ demonstrated that the quality of *S.* *hortensis* essential oil is increasing during storage, for example, the carvacrol content of the sample increased from 50.69% to 57.66% when the sample was kept for six months in the freezer (at -20 ºC).

TG/DTG/DTA is a useful thermo-analytical technique to investigate the mass loss of a sample as a function of time or temperature. The decomposition temperature corresponding to the maximum slopes of each mass-loss stage recorded on the ThermoGravimetric curve is seen as a peak when the Derivative ThermoGravimetric (DTG) curve is plotted^17^. This technique was used to characterize the thermal stability of some essential oils, i.e. orange, lemongrass, and basil oil^15^, as well as to evaluate the activation energy for the evaporation of lavender, cypress, tea tree, clove, eucalyptus, etc. oils^13,16^.

This study aimed to analyze the thermal behavior, by characterizing the recorded mass-loss stages and by evaluating the kinetic parameters of non-activated vaporization processes, for *S.* *hortensis* essential oil samples (heated and unheated) in correlation with their chemical composition and in vitro antioxidant activity. The correlation of the thermal behavior with the chemical composition for *S.* *hortensis* essential oil depending on the heating conditions treatment, to which the samples were subjected, constitute a novelty in the field.

## Results

### GC–MS analysis

**Figure 1:**
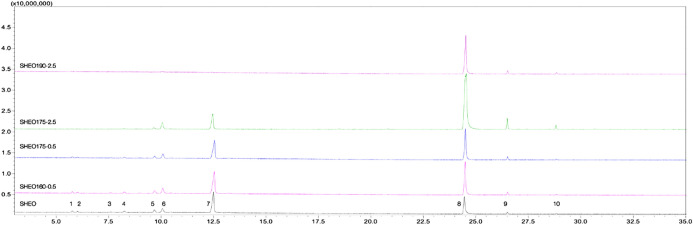
Chemical composition of *Satureja* *hortensis* essential oil. Unheated and heated essential oils chromatograms (1-α-thujene, 2-α-pinene, 3-sabinene, 4-β-pinene, 5-α-terpinolene, 6-*p*-cymene, 7-γ-terpinene, 8-carvacrol, 9-caryophyllene, 10-β-bisabolene) obtained by GC–MS. Annotations. *Satureja* *hortensis* essential oil denoted as SHEO and heated essential oils at different temperatures and periods denoted as SHEOx-y, where x is the temperature of treatment (ºC), and y is the heating time in hours (SHEO160-0.5, SHEO175-0.5, SHEO175-2.5, and SHEO190-2.5).

The chemical composition of the unheated and heated essential oils obtained from *S.* *hortensis* has been determined using GC–MS analysis, and the chromatograms are depicted in Fig. 1.

### UHPLC-MS analyses

**Figure 2:**
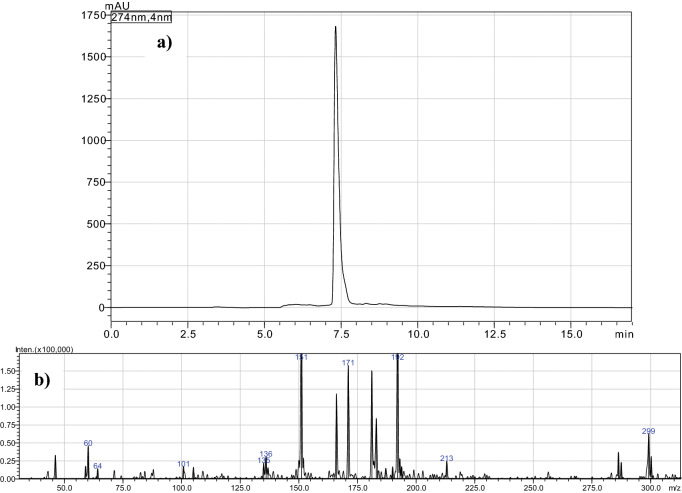
The chromatogram obtained by RP-UHPLC for the essential oil obtained from *S.* *hortensis* heated for 2.5 h at 190 ºC (SHEO190-2.5), **(a)**, and mass spectrum recorded for the compound found at 7.32 min **(b)**.

The reverse-phase ultra-liquid chromatography coupled with mass spectrometry (RP-UHPLC-MS) analyses were performed to evaluate the changes of the essential oil composition, for non-volatile compounds, before and after heating treatment. Figure 2a is depicted the chromatogram, and in Fig. 2b is the mass spectrum recorded for the heated essential oil sample SHEO190-2.5.

### In vitro antioxidant activity

The antioxidant activity of the samples was determined using radical scavenging DPPH (1,1-diphenyl-2-picrylhydrazyl) free assay, as earlier reported^24^. The data obtained revealed that for the *S.* *hortensis* essential oil (SHEO), the inhibition is 80.02 ± 0.24%, while for the heated essential oil sample (SHEO190-2.5), inhibition was 87.73 ± 0.08%.

### ATR-FTIR analysis

**Figure 3:**
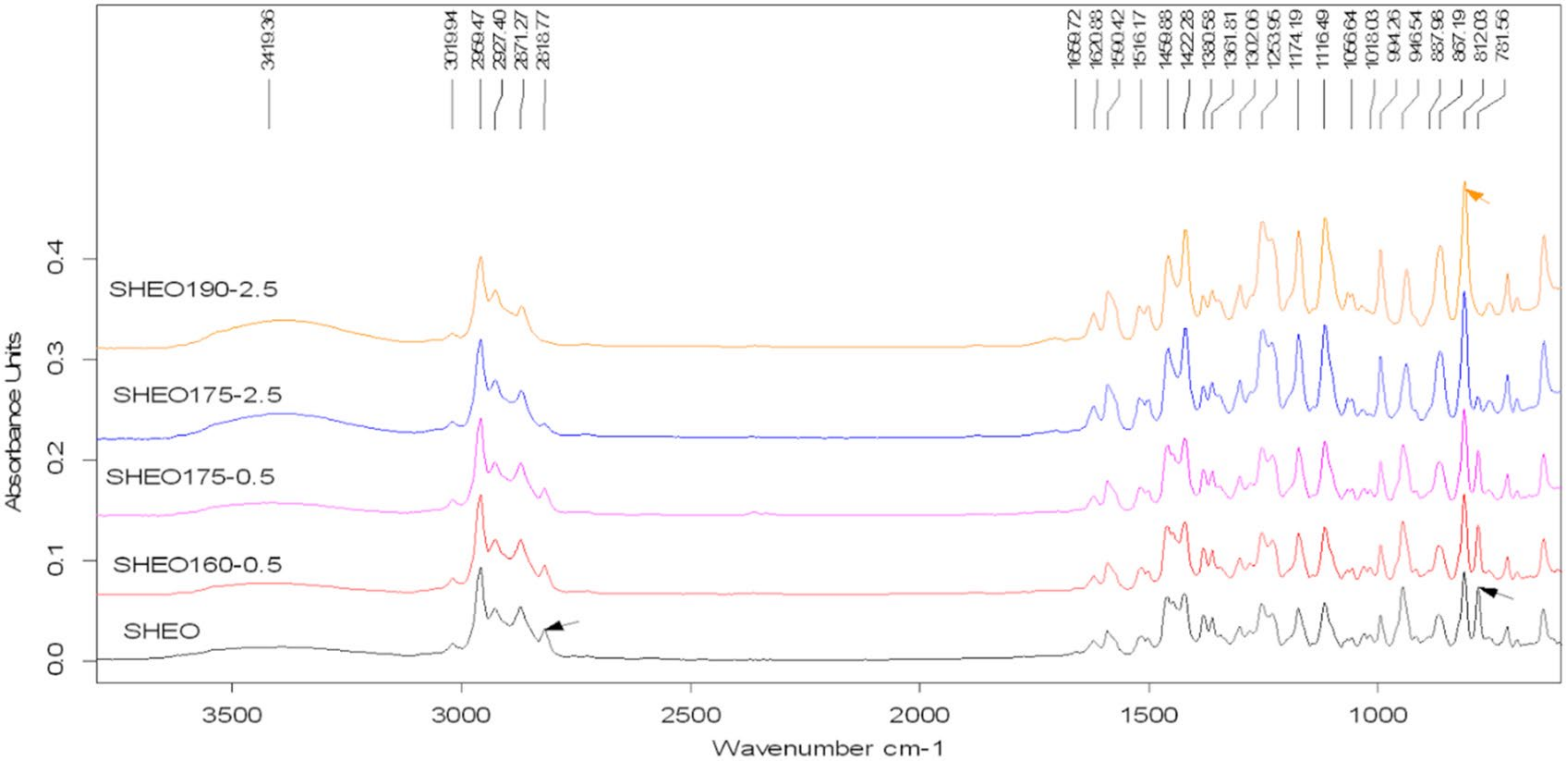
ATR-FTIR spectra recorded for *S.* *hortensis* essential oil samples. Annotations are as for Fig. 1.

The Attenuated Total Reflection Fourier-Transform Infrared (ATR-FTIR) spectra for all the samples were recorded to better elucidate the changes in the chemical composition of the essential oil during the heating treatment (Fig. 3). There was identified an intense band at 812 cm^−1^, and other bands at 867 cm^−1^, 994 cm^−1^, 1116 cm^−1^ 1174 cm^−1^, 1459 cm^−1^, 1422 cm^−1^, 1302 cm^−1^, 1253 cm^−1^, and 3200–3600 cm^−1^ from carvacrol ^25^.

Another signals in ATR-FTIR spectra were located at 781 cm^−1^, 813 cm^−1^, 873 cm^−1^, 886 cm^−1^, 946 cm^−1^, 1056 cm^−1^, 1018 cm^−1^, 1380 cm^−1^, 1361 cm^−1^ 1516 cm^−1^, 1590–1659 cm^−1^, 2818 cm^−1^, 2871 cm^−1^, 2959 cm^−1^, 2972 cm^−1^, and 3019 cm^−1^.

### Thermal analysis

**Figure 4:**
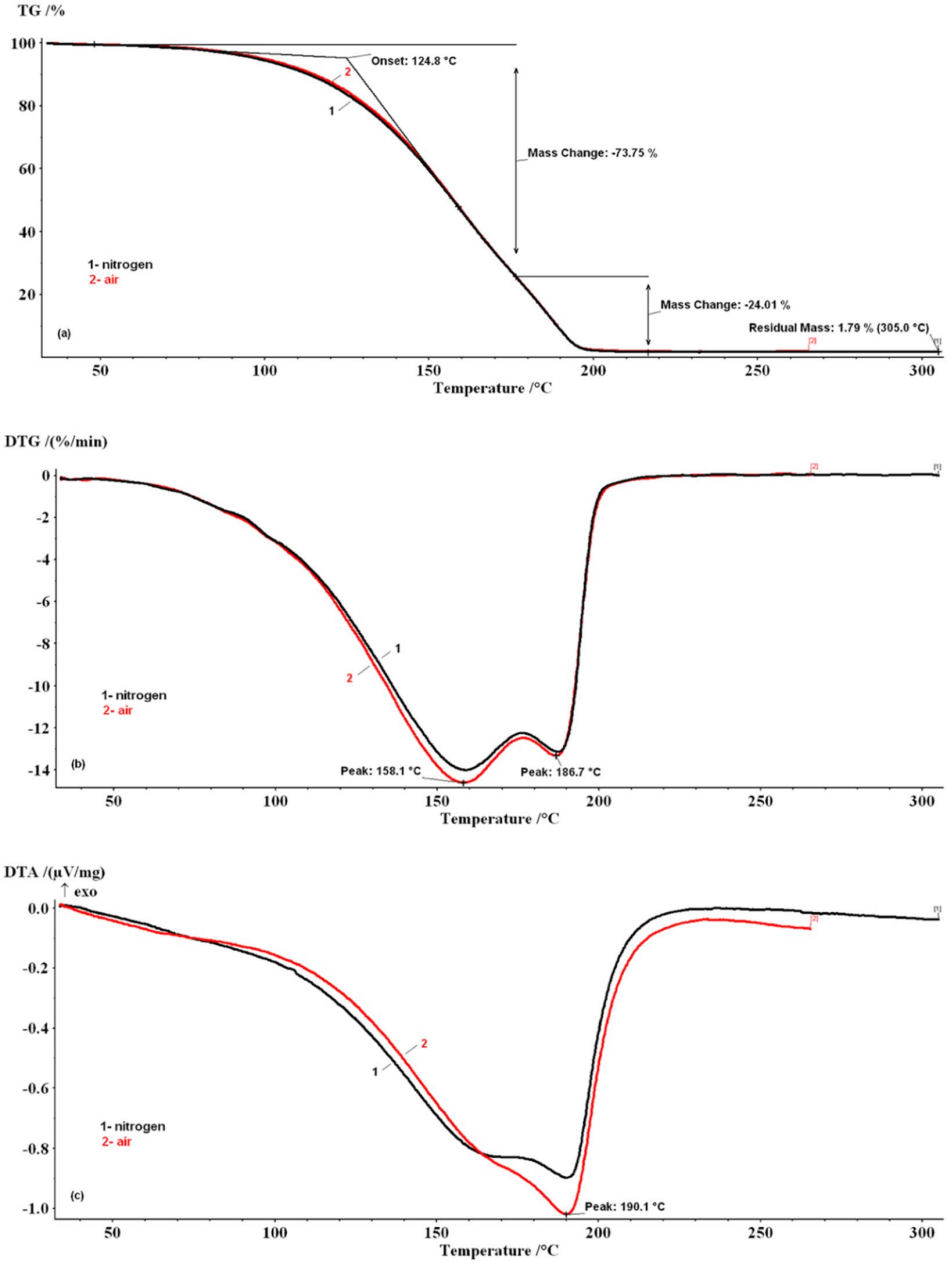
Thermo-analytical curves of SHEO in the nitrogen and air decomposition atmosphere. **(a) **TG curves; **(b)** DTG curves; **(c)** DTA curves.

**Figure 5:**
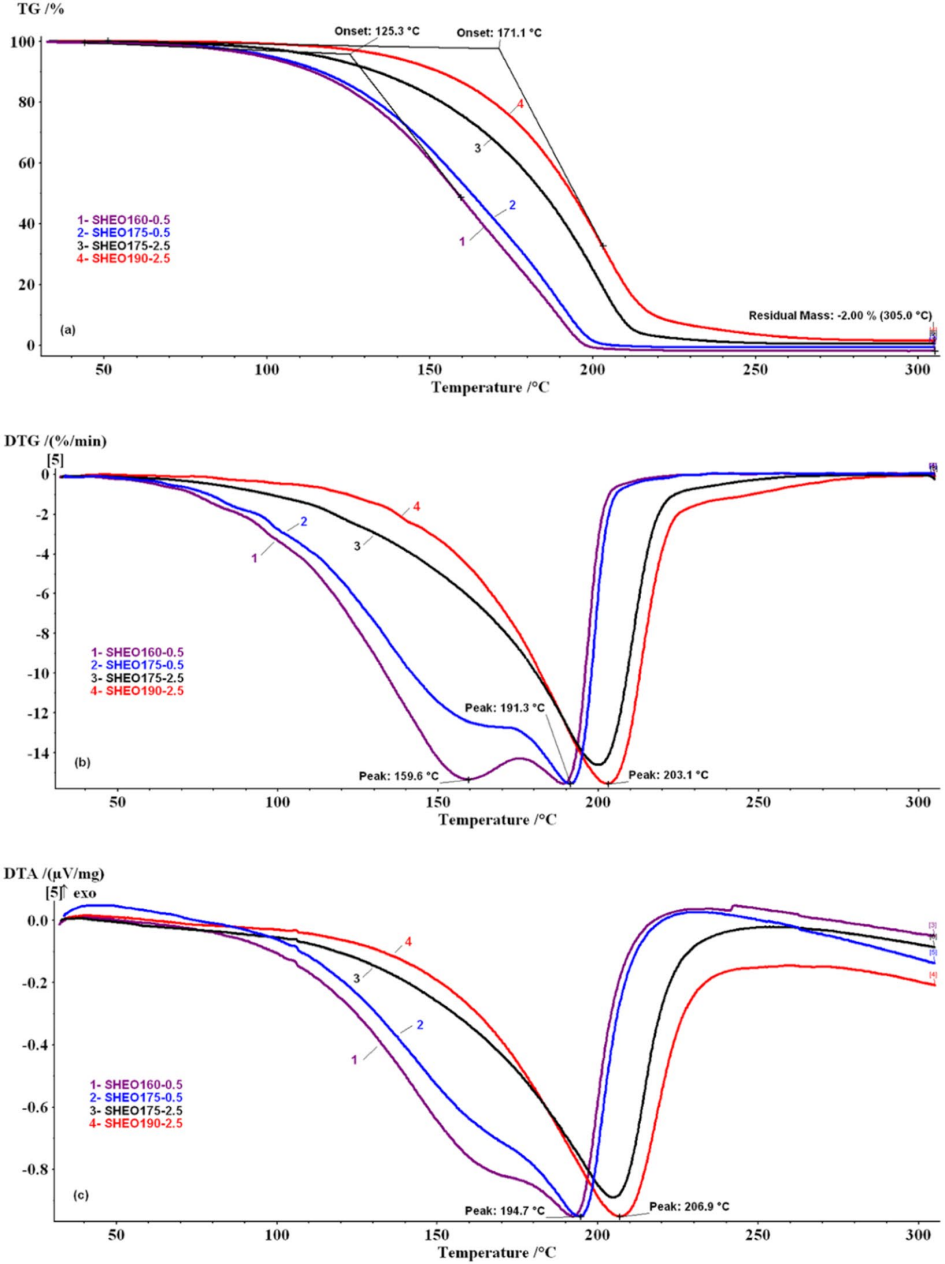
Thermo-analytical curves of SHEOx-y samples in the nitrogen decomposition atmosphere. **(a)** TG curves; **(b) **DTG curves; **(c)** DTA curves.

The TG/DTG/DTA curves of the essential oil samples were recorded in air, and the nitrogen decomposition atmosphere, and are depicted in Figs. 4 and 5. For the SHEO sample, the thermal stability between 30 and 50 ºC is visible, while in the 50–220 ºC temperature range, TG curves show a continuous mass-loss. In Fig. 5 are presented DTG and DTA curves for the samples with two peaks for SHEO160-0.5 and SHEO175-0.5 samples, and only one peak for SHEO175-2.5 and SHEO190-2.5 samples.

## Discussion

*Satureja* *hortensis* essential oil (SHEO) revealed a chemical composition with 18 constituents identified, rich in monoterpenes (96.99%) and sesquiterpenes (3.01%) by GC–MS analyses. As presented in Table 1, SHEO’s major components were: γ-terpinene (42.30%), carvacrol (32.83%), *p*-cymene (8.05%), α-terpinolene (5%), β-pinene (2.32%), caryophyllene (2.22%), α-thujene (1.93%), α-pinene (1.49%), cis-sabinene hydrate (0.87%), β-bisabolene (0.79%), limonene (0.77%), sabinene (0.66%), α-phellandrene (0.63%), carvacrol methyl ether (0.09%) and four detected minor components: terpinen-4-ol, isocaryophyllene, cis-α-bisabolene, and caryophyllene oxide.

**Table 1:** Chemical composition, as determined by GC–MS, of unheated and heated *S.* *hortensis* essential oil samples with retention Kovats indices (KI) calculated in this study and reported in literature^27^ (*Satureja* *hortensis* essential oil was denoted as SHEO and heated essential oils at different temperatures and periods indicated as SHEOx-y, where x is the temperature of treatment (ºC), and y is the heating time in hours (SHEO160-0.5, SHEO175-0.5, SHEO175-2.5, and SHEO190-2.5).

Nr. Crt	KI calc/lit	Compound	SHEO (%)	SHEO160-0.5 (%)	SHEO175-0.5 (%)	SHEO175-2.5 (%)	SHEO190-2.5 (%)
1	930/930	⍺-Thujene	1.93	1.47	0.96	0.18	n.d
2	939/939	⍺-Pinene	1.49	0.94	0.79	0.19	n.d
3	979/975	Sabinene	0.66	0.71	0.47	n.d	n.d
4	983/979	β-Pinene	2.32	1.72	1.43	0.26	n.d
5	1010/1002	α-Phellandrene	0.63	0.32	n.d	n.d	n.d
6	1082/1088	α-Terpinolene	5	3.8	3.29	1.08	n.d
7	1098/1091	*p*-Cymene	8.05	7.32	7.71	5.49	1.33
8	1014/1029	Limonene	0.77	0.43	n.d	n.d	n.d
9	1066/1059	γ-Terpinene	42.35	36.19	34.82	12.85	n.d
10	1073/1070	*cis*-sabinene hydrate	0.87	0.79	0.58	n.d	n.d
11	1180/1177	Terpinen-4-ol	t	0.36	0.05	0.41	n.d
12	1250/1244	Carvacrol methyl ether	0.09	0.23	0.26	0.32	n.d
13	1295/1299	Carvacrol	32.83	42.3	45.73	71.27	91.36
14	1415/1408	β-Caryophyllene	2.22	2.43	2.88	5.24	4.97
15	1510/1505	β-Bisabolene	0.79	0.32	0.27	0.16	2.34
16	1521/1419	Isocaryophyllene	t	0.67	0.76	1.91	n.d
17	1516/1507	*cis*-α-bisabolene	t	t	t	0.15	n.d
18	1587/1583	Caryophyllene oxide	t	t	t	0.49	n.d

The chemical composition of essential oil samples unheated or subjected to heating treatment at different temperatures (160, 175, 190 ºC) and periods (0.5 and 2.5 h, respectively), is entirely different. In SHEO the principal components were γ-terpinene, carvacrol, *p*-cymene, and *α*-terpinolene, while for SHEO190-2.5 carvacrol is the major component. Depending on temperature and heating time, γ-terpinene content in the mixture had decreased linearly from 42.35% to 0%, while, carvacrol concentration increased from 32.83% to 91.36%.

Starting with SHEO175-0.5, some components volatilized completely (α-phellandrene, limonene), while more thermal resistant components began to increase. In the case of SHEO175-2.5, the changes are greater, more components volatilized (sabinene, cis-sabinene hydrate) while previously detected components are now quantified (cis-α-bisabolene and caryophyllene oxide). For SHEO190-2.5 only stable thermal components remained: *p*-cymene (C_10_H_14_, b.p. 177 ºC), carvacrol (C_10_H_14_O, b.p. 237.7 ºC), caryophyllene (C_15_H_24_, b.p. 262–264 ºC) and β-bisabolene, (C_15_H_24_, b.p. 275 ºC)^26^ while all others were evaporated.

It was evident that we cannot determine by HPLC the volatile compounds identified by GC–MS in the present study, such as γ-terpinene, carvacrol, *p*-cymene, caryophyllene, β-bisabolene, as also was reported by Rainis and Ternes^28^ for *Thymus* *vulgaris* extract. Instead, for SHEO190-2.5 we obtained only one major peak at 7.32 min with a concentration higher than 95.5%, as is depicted in Fig. 2. The mass spectrum for this compound presented the following *m/z* values: 299(23), 286(13), 192(100), 183(30), 181(55), 171(58), 166(43), 151(83), 136(11), 105(6), 101(6), 60(17), 46(12). The analysis of the mass spectrum pattern leads to the conclusion that the main compound observed is the dimer of carvacrol (C_10_H_14_O, M = 150 Da) in the SHEO190-2.5 sample, the compound obtained by the dimerization of carvacrol from the sample due to the method used for this separation. As was confirmed by GC–MS analysis, in SHEO190-2.5 carvacrol is the major compound in the sample.

The in vitro antioxidant activity of SHEO and SHEOx-y samples was assessed using DPPH as a free radical, measuring the capacity of these essential oils to donate H or e^-^. The data shown inhibition varying from 80 to 87.7% for the analyzed samples, which implicates remarkable antioxidant activity. The higher inhibition was observed for the heated essential oils (SHEO190-2.5), which presents the highest level of carvacrol 87.73 ± 0.08% compared with no treated oil (SHEO) 80.02 ± 0.24%. The phenolic hydroxyl group, which is present in carvacrol is a good H donor and can react with reactive oxygen species disabling the reaction of making newer radical generations^29^. It was observed that with higher inhibition, there is an improvement in the thermal stability of the oil and a higher value of T_onset_. Therefore, T_onset_ for unheated essential oil was at 124.8 ºC, and after the heating treatment, T_onset_ got up to 171.1 ºC, making the essential oil more antioxidant and increase the thermal stability.

According to GC–MS results, in the case of SHEO, the compounds which have a major influence on the ATR-FTIR spectrum are *γ*-terpinene, carvacrol, and *p*-cymene. A small influence could be given by α-terpinolene, α-pinene, β-pinene, and caryophyllene. The recorded ATR-FTIR spectra for the investigated samples are depicted in Fig. 3 and several characteristic bands can be extracted even if the analyzed essential oil is a complex mixture and the spectra display a total overlap of each absorption spectrum of its components. The intense band seen at 812 cm^−1^ can be attributed to out-of-plane C–H waggins vibrations from carvacrol. This significant signal is used in distinguishing the type of aromatic ring substitution in the isomeric compounds carvacrol and thymol, with the thymol isomer presenting its vibration at 804 cm^−1^ ^25,30,31^. Furthermore, other characteristic bands of carvacrol were identified at: 867 cm^−1^ (C–H out-of-plane bending vibration), 994 cm^−1^ (wagging vibration of 1:2:4 substitution), 1116 cm^−1^ (wagging vibration of *ortho*-substitution) and 1174 cm^−1^ (*ortho*-substitution), 1459, 1422 and 1302 cm^−1^(C–C stretching vibration from phenyl group and CH_2_ bending vibration), 1253 cm^−1^ (C-O stretching vibration)^25,30,31^. At 3200–3600 cm^−1^, the -OH stretching vibration from carvacrol was observed^25^. The corroboration of the ATR-FTIR results and the GC–MS data allow us to denote that carvacrol is the main monoterpenic phenol isomer in SHEO.

Another major component in the SHEO composition, identified in the GC–MS analysis is *γ-*terpinene which presents the characteristics signals in ATR-FTIR spectra located at 781 cm^−1^due to C–H out of plane bending (strong), 946 cm^−1^ due to C–C stretching (strong to weak), and 2818 cm^−1^ due to C–H stretching (medium to strong) ^32^. Most of the terpenoids show also wagging vibrations of C–H and CH_2_ groups, e.g. *p*-cymene at 813 and 1516 cm^−1^ ⍺-terpinolene at 781 cm^−1^, α-pinene at 886 cm^−1^, β-pinene at 873 cm^−1^, caryophyllene at 885 cm^−1^ ^33^. For *p*-cymene, the other two characteristics bands were recorded for SHEO at 1056 and 1018 cm^−1^, respectively, that were assigned to *para*-substitution ^33^. Some bands recorded in the ATR-FTIR spectra of SHEO are common to the major constituents of the oil. Thus, bands at 1380 and 1361 cm^−1^ are due by symmetric and asymmetric bending of isopropyl groups^33^, and the signal from 1516 cm^−1^ is due by waging vibration of C–H(CH_3_) ^25^. The bands located between 1590–1659 cm^−1^ are due by C=C stretching vibration ^25^ while those at 2871, 2972, 2959, and 3019 cm^−1^ were assigned to C–H stretching vibrations specific to CH_3_^30^.

Comparing the spectra of SHEO and SHEOx-y samples, it can be noted that as the temperature and time heating increase, significant changes are occurring. The characteristic bands of terpinene isomers (γ-terpinene + α-terpinolene) decrease in intensity while the characteristic carvacrol and *p*-cymene bands amplify (Fig. 3).

In the case of SHEO190-2.5, the total disappearance of the bands at 781, 946, and 2818 cm^−1^ was found, while the band at 812 cm^−1^ is given by out-of-plane C–H wagging vibrations, and the band recorded between 3200–3600 cm^−1^ provided by OH stretching vibration, increase in intensity. Regarding the *p*-cymene specific bands, these decreases in intensity, suggest that after 190 ºC, the main remaining component of essential oil is carvacrol.

According to Siqueira, et al. ^14^, the thermal resistance or susceptibility of the essential oils may be correlated to their chemical composition. From the GC–MS and FT-IR recorded data, it was observed that SHEO contains in similar proportions two categories of monoterpenes (~ 48% γ-terpinene + α-terpinolene and ~ 42% carvacrol + p-cymene). Most likely, this particular composition determines the two-recorded steps accompanied by mass-loss. The mass-loss steps recorded on TG/DTG/DTA curves for other plant essential oils were due to the evaporation processes, as was highlighted in previously published results ^14,15,17,19^. Thermo-analytical curves for SHEOx-y samples are (Fig. 4), and their characteristic thermal parameters are presented in Table 2. The TG/DTG/DTA curves of the SHEO sample recorded in air, respectively, in the nitrogen decomposition atmosphere are similar and show the stability of the sample between 30–50 ºC. In contrast, in the 50–220 ºC temperature range, TG curves present continuous mass-loss. The DTA profile curves suggest that the endothermic processes which take place are complex and involve at least two overlapped steps. These steps are better highlighted on the DTG curves.

**Table 2:** Thermal parameters from TG/DTG curves for SHEOx-y samples.

Sample	T_onset_ (K)	Step 50–175 ºC		Step 175–220 ºC		Step 50–220 ºC	
		T_DTG_ (K)	Mass-loss (%)	T_DTG_ (K)	Mass-loss (%)	T_DTG_ (K)	Mass-loss (%)
SHEO160-05	125.3	159.6	70.54	189.3	30.81	–	–
SHEO175-05	132.9	nd	55.56	191.3	44.76	–	–
SHEO175-2.5	165.4	–	–	–	–	200.0	99.2
SHEO190-2.5	171.1	–	–	–	–	203.1	98.2

The first step was recorded in both decomposition gases on the 50–175 ºC temperature range with a ~ 73.7% mass-loss. The maximum DTG peak was reached at T_DTG_ = 158.1 ºC. The second step was recorded between 175–220 ºC temperature range, with a mass-loss value of ~ 24.1% and a T_DTG_ = 186.7 ºC. For this stage, the DTG curves present an abrupt profile, returning from the maximum point to baseline. Similar behavior was also reported for orange, lemongrass, and basil essential oils by Martins, et al. ^15^ and for aromatherapy, essential oils by Chen, et al. ^13^ and was attributed to a zero-order vaporization process.

Besides, the T_onset_ values were recorded at 124.8 ºC in both working atmospheres, and a small residual mass of ~ 1.79% was noticed at ~ 300 ºC. These results imply that the decomposition flow types do not influence the thermal stability of the SHEO and the recorded mass-loss steps, because they are not determined by the thermo-oxidative or pyrolysis degradation reactions.

From Fig. 5 it can be observed that as the heating temperature and time increases, the shape of the DTG and DTA curves shows some changes compared to those obtained for the SHEO sample. Thus, if the SHEO160-0.5 sample still presents the two peaks on the DTG curve well separated, for the SHEO175-0.5 sample the DTG peak recorded in 50–175 ºC range becomes a shoulder and the peak recorded in the 175-220ºC field shows an increase to T_DTG_ = 191.3ºC. The DTA curves present an endothermic complex effect with a maximum that rises from T_DTA_ = 190.1ºC (SHEO) to 194.7ºC (SHEO175-0.5). For SHEO175-2.5 and SHEO190-2.5 samples, only one mass-loss step was recorded in 50–220 ºC temperature range with the T_DTG_ ~ 203.1 ºC and T_DTA_ ~ 206.9 ºC. Besides, the shape of DTA endothermic curves becomes more symmetrical.

In Table 2, significant changes are noticed for T_onset_ values compared with SHEO (T_onset_ = 124.8 ºC). These values increase from T_onset_ = 125.3ºC to 171.1ºC, which means that the thermal stability of SHEOx-y samples increases as a result of heating treatment, the heated samples being more stable than the SHEO sample. Compared with the SHEO sample, the SHEO160-0.5 and SHEO175-0.5 show two steps: decrease in mass-loss values in both steps: (i) in the 50–175 ºC range a decrease in mass-loss values to 70.54% and 55.56% respectively, and (ii) in the 175–220 ºC range an increase to 30.8% and 44.76% respectively. The variations observed in mass-loss values are probably due to the vaporization, in 50–175 ºC range, of the compounds less stable to heating, which leads to the enrichment of the essential oil samples in the more stable components. These become preponderant in SHEO175-2.5 and SHEO 190–2.5 samples for which only one stage of mass-loss of (~ 98–99%) was achieved.

All changes observed in the thermal behavior of SHEOx-y samples compared to SHEO are due to the changes that occurred in the chemical composition after heating, as can be seen from the GC–MS and FT-IR results. The decrease in *γ*-terpinene + *α*-terpinolene content observed in the GC–MS analysis as well as the diminishing of the specific bands intensity (2818, 946 and 781 cm^−1^) in the FT-IR spectra of SHEOx-y samples, demonstrates that after heating the samples to 175 ºC these monoterpenes are eliminated from the oil through vaporization. In the case of SHEO190-2.5, the total absence of the specific bands for γ-terpinene and its isomer α-terpinolene was observed while an significant increase of intensity for carvacrol specific bands took place.

Based on these observations, the first degradation step recorded in the TG/DTG/DTA curves for SHEO between 50–175 ºC can be due to vaporization mainly of γ-terpinene + α-terpinolene and to a small fraction of carvacrol + *p*-cymene, while the second step, recorded between 175–220 ºC, can be attributed to the vaporization of the remained major fraction of carvacrol. Regarding the *p*-cymene compound, this vaporizes on the 175–220 ºC range but at a lower temperature than 190 ºC.

For a step recorded in the TG curve to be given by a non-activated vaporization process, the mass-loss recorded relative to time or temperature should be a zero-order transformation. According to Hazra, et al. ^34^ and Martins, et al. ^15^, the shape of the DTG curve has an essential role in deciding the reaction order. Thus, for zero-order kinetic, the DTG curve is characterized by an abrupt return from the maximum point (*T*_*DTG*_) to the baseline.

**Figure 6:**
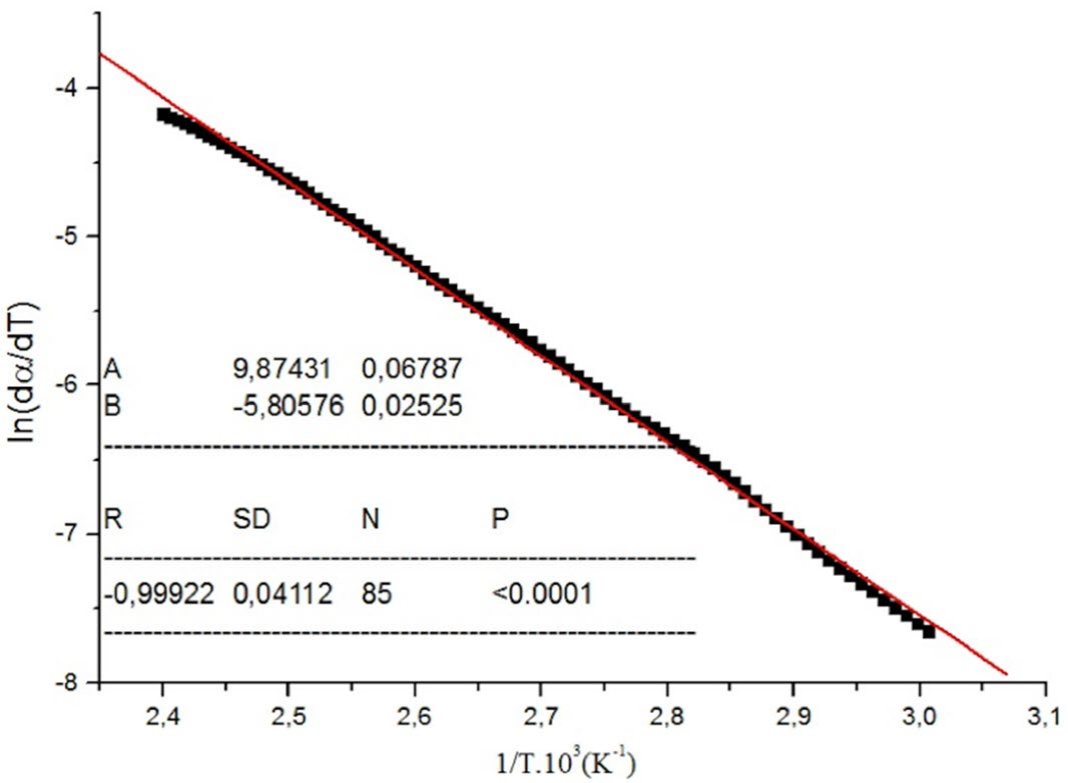
Arrhenius plot for SHEO samples decomposed air atmosphere.

All essential oil samples investigated in the present work showed this behavior of the DTG curves, and this can be seen in Figs. 3 and 4. Aiming to determine their vaporization kinetic parameters, we considered this process to be a zero-order. The kinetic vaporization parameters obtained for SHEO in air/nitrogen flow and SHEOx-y in nitrogen flow, as well as a typical Arrhenius plot used for the *E*_*a*_ calculation, are shown in Fig. 6 and Table 3. As the vaporization of γ-terpinene + α-terpinolene from the samples progresses, it can be observed that, in the range of 50–175 °C, the *E*_*a*_ values increase (from ~ 45 kJ mol^−1^ to ~ 49.6 kJ mol^−1^) with the rise in the samples thermal stability and the decrease of mass-loss values. For the SHEO175-0.5 and SHEO190-2.5 samples, the *E*_*a*_ values characterize the vaporization process of the carvacrol and *p*-cymene, which are more stable to heating.

**Table 3:** Vaporation kinetic parameters of SHEO and SHEOx-y samples, where: *r*^2^ is the linear correlation coefficient.

Parameters	SHEO air	SHEO nitrogen	SHEO160-0.5	SHEO175-0.5	SHEO175-2.5	SHEO190-2.5
	50–175 ºC				50–220 ºC	
*E*_*a*_ (kJ mol^−1^)	45.12	45.2	48.12	49.69	55.67	57.12
*A* (s^−1^)	3.1 × 10^10^	1.1 × 10^10^	6.3 × 10^9^	7.9 × 10^10^	6.3 × 10^7^	6.3 × 10^11^
*r*^2^	0.9992	0.9994	0.9991	0.9991	0.9998	0.9996

*E*_*a*_ values are in good agreement with those reported for other essential oils, i.e., basil oil: 39.63 kJ·mol^−1^ ^15^ lavender oil: 48.15 kJ·mol^−1^ ^13^, cinnamon oil: 51.0 kJ·mol^−1^ ^16^. The data corroborated in the present study suggest that a rapid route to obtain carvacrol is the heating of the essential oil of *S.* *hortensis* at 190 ºC, a temperature below the boiling point of the compound.

In this study, *Satureja* *hortensis* essential oil (SHEO), and a series of heated samples at different temperatures and periods (SHEOx-y) were investigated by GC–MS, HPLC–MS, ATR-FTIR, and TG/DTG/DTA to characterize the heating conditions influence on the chemical composition and thermal behavior. For the SHEO sample, the GC–MS analysis showed that the major constituents of chemical composition were γ-terpinene + α-terpinolene (~ 48%) and carvacrol + *p-*cymene (~ 42%), compounds whose characteristic absorption bands predominate the ATR-FTIR spectrum of the sample. TG curves recorded on air or nitrogen atmosphere show a continuous mass-loss in 50–220 °C range. In contrast, the DTA profile curves suggest that the endothermic processes which take place are complex and involve at least two overlapped mass-loss steps better highlighted on the DTG curves. Based on the obtained results the first degradation step could be due to vaporization mainly of γ-terpinene + α-terpinolene and to a small fraction of carvacrol + *p*-cymene, while the second step, recorded between 175–220 ºC, could be attributed to vaporization of the remained major fraction of carvacrol and *p*-cymene. For the SHEOx-y samples, it can be noted that as the temperature and heating time increase, major changes in chemical composition are occurring. In the ATR-FTIR spectra a decrease up to the total absence of the terpinene isomers (γ-terpinene + α-terpinolene) characteristic bands were observed, while those for carvacrol and *p*-cymene amplified. The TG/DTG/DTA curves showed a decrease in mass-loss values for the 50–175 ºC step and an increase in 175–220 ºC range. For the SHEO175-2.5 and SHEO 190–2.5 samples, only one mass-loss stage was achieved. As the vaporization of γ-terpinene + α-terpinolene from samples progresses, an increase in the activation energy (*E*_*a*_) values with the increase of the samples' thermal stability (T_onset_) was observed. Corroborating all the data obtained, the heating of *Satureja* *hortensis* essential oil at 190 ºC for two and a half hours lead to the obtaining of carvacrol as the main component. This study demonstrated the importance of analyzing the chemical composition of essential oils after thermal exposure with essential effects on the uses of the essential oils as a natural preservative for the food industry and aromatherapy.

## Materials and methods

### Plant material

Aerial parts (stems with leaves and flowers) of *S.* *hortensis* were gathered in the summer of 2017 when at least 50% of the flowers were in bloom, and the volatile oil content reached its maximum. The plants were harvested in late July from a local producer (21º 19′ E longitude and 46º 9′ N latitude), Arad county. Harvested plant material was dried at 35ºC for 7 days using a drying oven (Model FD23, Binder, Germany), and voucher specimens were taken and stored at the Institute of Technical and Natural Sciences Research-Development-Innovation of "Aurel Vlaicu" University of Arad.

### Essential oil extraction

Aerial parts of *S.* *hortensis* were separated into two categories: stalks and leaves + flowers. Only the leaves and flowers mix was subjected to steam distillation using a 5 L copper alembic distillation equipment, and the extraction yield was ~ 2.5%.

### Preparation of SHEO heated samples

The SHEOx-y samples were obtained from SHEO (1 mL) through different heating temperatures (160, 175, 190 ºC) and periods (0.5 and 2.5 h) in open glass vials using a drying oven (Model FD23, Binder, Germany) with temperature control. The mass loss determined for the essential oil during heat treatment, at 160 °C, 175 °C, and 190 °C, respectively is 31.17%, 41.43%, 96.10% after 30 min, and 84.33%, 96.85%, and 99.79% after 2.5 h. We chose these three heating tempratures as we classified the compounds determined in the essential oil in three classes: a) compounds with boiling point < 160 °C, b) compounds with boiling point 160–190 °C; c) compounds with boiling point > 190 °C. Also we chose two periods of heating: 0.5 h and 2.5 h to observe the time-related behavior of the essential oil.

### Free radical scavenging activity (DPPH assay)

For the analysis, 3 mL ethanolic solution of DPPH (0.4 mM) was placed in glass vials, and to that 20 µL of SHEO and SHEOx-y, respectively, were added. Spectrophotometric analysis was performed using the radical DPPH as reference, and results were expressed as inhibition (%).

The readings were carried out after 1 h of incubation, in the dark, at room temperature, with a ScanDrop Nano-volume Spectrophotometer from Analytik Jena (Germany) at 517 nm using a 10 mm quartz cuvette. The percentage of DPPH inhibition was calculated with Eq. (1):

1 $$\%Inhibition=\left(\frac{{Abs}_{control}-{Abs}_{sample}}{{Abs}_{control}}\right)x 100$$

where: Abs control is the absorbance of 0.4 mM DPPH in ethanol, and Abs sample is the absorbance of 0.4 mM DPPH containing essential oil after 1 h.

### GC–MS analysis

SHEO constituents were determined by a gas chromatograph (Shimadzu2010, Kyoto, Japan) coupled with a triple quadrupole mass spectrometer (TQ 8040, Shimadzu, Kyoto, Japan). The used column was an optima 1MS + WAX column (30 m × 0.25 mm i.d., 0.25 µm film thickness, Macherey–Nagel, Duren, Germany) with He as a carrier gas and a 1 mL min^−1^ flow. The oven temperature was initiated at 70 ºC for 11 min, and raised to 190 ºC at a rate of 5 ºC min^−1^ and then to 240 ºC at a rate of 20 ºC min^−1^ where it was left for 5 min. Injector and MS source temperatures were set to 250 ºC and 200 ºC, respectively. The injection volume was 1 µL, with a split ratio of 10:1. SHEO constituents have been identified based on their mass spectra using the NIST 14 library and Wiley 09 library.

### UHPLC-MS analyses

The analyses were performed using a liquid chromatograph (Nexera X2, Shimadzu, Tokyo, Japan) with a diode array detector (M30A, Shimadzu, Tokyo, Japan) and a mass spectrometer (Model 8040, Shimadzu, Tokyo, Japan). The separation of compounds was performed on column (Nucleosil EC 135/4 100–3 C18, 4.0 mm i.d. × 125 mm column length, 3 µm particle size, Macherey–Nagel GmbH, Duren, Germany). The column temperature was maintained at 25 ºC, and the flow rate at 0.7 ml min^−1^. The solvents used for the chromatographic elution were ultra-pure water with 0.1% TFA (A) and acetonitrile (B). The chromatographic elution program as following: with 80% A and 20% B for 15 min, changed to 100% B in one minute, and then maintained at 100% B for another one minute. The injected volume of samples was 10 µl. The DAD detector spectra were recorded between 190 and 700 nm. The mass spectrometer was equipped with an electrospray ionization (ESI) source operated in positive ion mode, and quantification was carried out in the multiple reaction monitoring (MRM) mode. The mass range was between *m/z* 15 and 1500, scan speed 1500 u/sec. The ion spray temperature was maintained at 250 °C. The drying gas flow rate was 15 L/min.

### ATR-FTIR analysis

The FT-IR spectra of essential oil samples were recorded using Bruker Vertex 70 spectrophotometer equipped with the ATR cell, on the 600–4000 cm^−1^ wavelength range with a resolution of 4 cm^−1^ and 32 scans. For each analyzed sample, a background measurement was performed. The spectra were processed using OPUS software. The recorded FT-IR spectra were normalized (min–max), and the baseline-corrected.

### Thermal analysis

The TG/DTG/DTA thermo-analytical curves were recorded on an STA 409C Luxx system, produced by Netzsch-Germany. The experiments were conducted on 30-300ºC temperature range, at β = 10 K^·^min^−1^ heating rates, using platinum crucibles in dynamic air (100 mL.min^−1^, 20% O_2_) or a nitrogen atmosphere (100 mL·min^−1^, 99.99% N_2_). The sample mass was ~ 10 mg. The curves were processed using the Netzsch Proteus software.

### Kinetics of vaporization

The obtained TG/DTG data related to samples mass decrease in correlation with temperature was used to calculate the kinetics parameters (*A* is the frequency factor, *E*_*a*_ is the activation energy) and the calculus was performed using the following kinetic Eq. (2):

2 $$\frac{d\alpha }{{dt}}\, = \,k\left( {1\, - \alpha } \right)^{n}$$

where: *⍺* is the amount of vaporized sample, *t* is the time, *n* is the apparent reaction order, *k* is the vaporization constant rate.

It is well known that *k* depends on the temperature following the Arrhenius equation, which can be given in Eq. (3):

3 $$k_{vap} \, = \,Ae^{{ - \,\frac{{E_{a} }}{RT}}}$$

where: *E*_*a*_ is the activation energy of vaporization (kJ.mol^−1^), *A* is the frequency factor (s^−1^), *R* is the gas constant, *T* is the absolute temperature.

From Eqs. (2) and (3), and taking the natural logarithm, the following expression is obtained, shown in Eq. (4):

4 $$\ln \left( {\frac{d\alpha }{{dt}}} \right)\, = \,\ln A\left( {1 - \alpha } \right)^{n} \, - \,\frac{{E_{a} }}{RT}$$

Taking into account the heating rate equation, β = dT/dt, the Eq. (4) can be written as follows, in Eq. (5):

5 $$\ln \left( {\frac{d\alpha }{{dT}}} \right)\, = \,\ln \frac{A}{\beta }\left( {1 - \alpha } \right)^{n} \, - \,\frac{{E_{a} }}{RT}$$

For the vaporization process, the zero-order reaction (*n* = 0) is available, according to^15^ and^35^, therefore Eq. (6) can be addressed as follows:

6 $$\ln \frac{d\alpha }{{dT}}\, = \,\ln \frac{A}{\beta }\, - \,\frac{{E_{a} }}{RT}$$

TG experimental data can be input to Eq. (6) to obtain a linear relationship between ln(d⍺/dT) and 1/T. The slope and intercept of the Arrhenius plot were used to calculate *E*_*a*_ and *A* apparent kinetics parameters.

## References

[1] Zhou, L., Xu, J. & Peng, Y. in *Encyclopedia**of**Agriculture**and**Food**Systems* (ed Neal K. Van Alfen) 223–230 (Academic Press, 2014).

[2] Do TKT, Hadji-Minaglou F, Antoniotti S, Fernandez X (2015). Authenticity of essential oils. TrAC Trends Anal. Chem..

[3] Raut JS, Karuppayil SM (2014). A status review on the medicinal properties of essential oils. Ind. Crop. Prod..

[4] Stahl-Biskup, E. in *Medicinal**and**Aromatic**Plants—Industrial**Profiles* Vol. 24 (ed Ephraim Philip Lansky) 75–124 (CRC Press, 2003).

[5] Hassanzadeh, M. K., Tayarani Najaran, Z., Nasery, M. & Emami, S. A. in *Essential**Oils**in**Food**Preservation,**Flavor**and**Safety* (ed Victor R. Preedy) 757–764 (Academic Press, 2016).

[6] Ali B (2015). Essential oils used in aromatherapy: A systemic review. Asian Pac. J. Trop. Biomed..

[7] Farzaneh, M., Kiani, H., Sharifi, R., Reisi, M. & Hadian, J. Chemical composition and antifungal effects of three species of Satureja (*S.**hortensis,**S.**spicigera,**and**S.**khuzistanica*) essential oils on the main pathogens of strawberry fruit. *Postharvest**Biol.**Tech.* **109**, 145–151, 10.1016/j.postharvbio.2015.06.014 (2015).

[8] Jafari F, Ghavidel F, Zarshenas MM (2016). A critical overview on the pharmacological and clinical aspects of popular *Satureja* species. J. Acupunct. Meridian Stud..

[9] Mohammadhosseini, M. & Beiranvand, M. Chemical composition of the essential oil form the aerial parts of *Satureja**hortensis* as a potent medicinal plant using traditional hydrodistillation. *J.**Chem.**Health**Risks* 43–54 (2013).

[10] Sharifzadeh, A., Khosravi, A. R. & Ahmadian, S. Chemical composition and antifungal activity of *Satureja**hortensis* L. essentiall oil against planktonic and biofilm growth of Candida albicans isolates from buccal lesions of HIV+ individuals. *Microb.**Pathog.* **96**, 1–9, 10.1016/j.micpath.2016.04.014 (2016). 10.1016/j.micpath.2016.04.014 27126187

[11] Dzida, K. *et**al.* Yield and quality of the summer savory herb (*Satureia**hortensis* L.) grown for a bunch harvest. *Acta**Sci.**Pol.**Hortorum**Cultus* **14**, 141–156 (2015).

[12] Nikolić M (2014). Chemical composition, antimicrobial, and cytotoxic properties of five Lamiaceae essential oils. Ind. Crops Prod..

[13] Chen W-T (2018). Structural characteristics and decomposition analyses of four commercial essential oils by thermal approaches and GC/MS. J. Therm. Anal. Calorim..

[14] Siqueira, G. L. D. A. D. *et**al.* Thermoanalytical evaluation of essential oils of the leaves from Eucalyptus spp susceptible and resistant to Glycaspis brimblecombei. *Braz.**J.**Therm.**Anal.* **5**, 10.18362/bjta.v5.i1.1 (2016).

[15] Martins, P., Sbaite, P., Benites, C. & Maciel, M. Thermal characterization of orange, lemongrass, and basil essential oils. *Chem.**Eng.**Trans.* **24**, 10.3303/CET1124078 (2011).

[16] Hazra A, Alexander K, Dollimore D, Riga A (2004). Characterization of some essential oils and their key components: Thermoanalytical techniques. J. Therm. Anal. Calorim..

[17] Barzegar, M., Ghaderi Ghahfarokhi, M., Sahari, M. A. & Azizi, M. H. Enhancement of thermal stability and antioxidant activity of thyme essential oil by encapsulation in chitosan nanoparticles*.**J.**Agric.**Sci.**Tech.* **18**, 1781–1792 (2016).

[18] Carrión-Prieto P (2017). Vibrational and thermal studies of essential oils derived from Cistus ladanifer and Erica arborea shrubs. Nat. Prod. Commun..

[19] Oliveira, C. E. L. D. & Cremasco, M. A. Determination of the vapor pressure of Lippia gracilis Schum essential oil by thermogravimetric analysis. *Thermochim.**Acta* **577**, 1–4, 10.1016/j.tca.2013.11.023 (2014).

[20] Fierascu, I. *et**al.* Phytochemical profile and biological activities of *Satureja**hortensis* L.: A review of the last decade. *Molecules* **23**, 10.3390/molecules23102458 (2018). 10.3390/molecules23102458 PMC6222901 30257512

[21] Sharifi-Rad M (2018). Carvacrol and human health: A comprehensive review. Phytother. Res..

[22] Granata G (2018). Essential oils encapsulated in polymer-based nanocapsules as potential candidates for application in food preservation. Food Chem..

[23] Mohtashami, S., Rowshan, V., Tabrizi, L., Babalar, M. & Ghani, A. Summer savory (*Satureja**hortensis* L.) essential oil constituent oscillation at different storage conditions. *Ind.**Crops**Prod.* **111**, 226–231, 10.1016/j.indcrop.2017.09.055 (2018).

[24] Csakvari AC (2019). Fatty acids profile and antioxidant activity of almond oils obtained from six romanian varieties. Farmacia.

[25] Valderrama ACS, De GCR (2017). Traceability of active compounds of essential oils in antimicrobial food packaging using a chemometric method by ATR-FTIR. Am. J. Anal. Chem..

[26] http://www.chemspider.com/.

[27] Adams, R. P. *Identification**of**Essential**Oil**Components**by**Gas**Chromatography/Mass**Spectrometry*. 4.1 edn (Allured Publishing, 2017).

[28] Rainis G, Ternes W (2014). Identification and characterization of dimeric oxidation products of p-cymene-2,3-diol isolated from *Thymus **vulgaris* L.. J. Agric. Food Chem..

[29] Pereira D, Valentão P, Pereira J, Andrade P (2009). Phenolics: From chemistry to biology. Molecules.

[30] Topala, C. M. & Tataru, L. D. ATR-FTIR study of thyme and rosemary oils extracted by supercritical carbon dioxide. *Rev.**Chim.-Bucharest* **67**, 842–846 (2016).

[31] Schulz H, Baranska M (2007). Identification and quantification of valuable plant substances by IR and Raman spectroscopy. Vib. Spectrosc..

[32] Coates, J. in *Encyclopedia**of**Analytical**Chemistry* (ed R. A. Meyers) 10815–10837 (Wiley, 2006).

[33] Schulz H, Özkan G, Baranska M, Krüger H, Özcan M (2005). Characterisation of essential oil plants from Turkey by IR and Raman spectroscopy. Vib. Spectrosc..

[34] Hazra A, Dollimore D, Alexander K (2002). Thermal analysis of the evaporation of compounds used in aromatherapy using thermogravimetry. Thermochim. Acta.

[35] Aggarwal P, Dollimore D, Alexander K (1997). The use of thermogravimetry to follow the rate of evaporation of an ingredient used in perfumes. J. Therm. Anal..

